# Evolution of Guanylate Binding Protein (*GBP*) Genes in Muroid Rodents (Muridae and Cricetidae) Reveals an Outstanding Pattern of Gain and Loss

**DOI:** 10.3389/fimmu.2022.752186

**Published:** 2022-02-09

**Authors:** João Vasco Côrte-Real, Hanna-Mari Baldauf, José Melo-Ferreira, Joana Abrantes, Pedro José Esteves

**Affiliations:** ^1^ Research Center in Biodiversity and Genetic Resources (CIBIO-InBIO), University of Porto, Vairão, Portugal; ^2^ Max von Pettenkofer Institute and Gene Center, Virology, National Reference Center for Retroviruses, Faculty of Medicine, Ludwig Maximilian University of Munich (LMU) München, Munich, Germany; ^3^ Department of Biology, Faculty of Sciences, University of Porto, Porto, Portugal; ^4^ BIOPOLIS Program in Genomics, Biodiversity and Land Planning, Research Center in Biodiversity and Genetic Resources (CIBIO), Vairão, Portugal; ^5^ Center of Investigation in Health Technologies (CITS), CESPU, Gandra, Portugal

**Keywords:** evolution, multigene family, GBP, innate immunity, muroids

## Abstract

Guanylate binding proteins (GBPs) are paramount in the host immunity by providing defense against invading pathogens. Multigene families related to the immune system usually show that the duplicated genes can either undergo deletion, gain new functions, or become non-functional. Here, we show that in muroids, the *Gbp* genes followed an unusual pattern of gain and loss of genes. Muroids present a high diversity and plasticity regarding *Gbp* synteny, with most species presenting two *Gbp* gene clusters. The phylogenetic analyses revealed seven different *Gbps* groups. Three of them clustered with *GBP2*, *GBP5* and *GBP6* of primates. Four new *Gbp* genes that appear to be exclusive to muroids were identified as *Gbpa*, *b*, *c* and *d*. A duplication event occurred in the *Gbpa* group in the common ancestor of Muridae and Cricetidae (~20 Mya), but both copies were deleted from the genome of *Mus musculus*, *M. caroli* and *Cricetulus griseus*. The *Gbpb* gene emerged in the ancestor of Muridae and Cricetidae and evolved independently originating *Gbpb1* in Muridae, *Gbpb2* and *Gbpb3* in Cricetidae. Since *Gbpc* appears only in three species, we hypothesize that it was present in the common ancestor and deleted from most muroid genomes. The second *Gbp* gene cluster, *Gbp6*, is widespread across all muroids, indicating that this cluster emerged before the Muridae and Cricetidae radiation. An expansion of *Gbp6* occurred in *M. musculus* and *M. caroli* probably to compensate the loss of *Gbpa* and *b*. *Gbpd* is divided in three groups and is present in most muroids suggesting that a duplication event occurred in the common ancestor of Muridae and Cricetidae. However, in *Grammomys surdaster* and *Mus caroli, Gbpd2* is absent, and in *Arvicanthis niloticus, Gbpd1* appears to have been deleted. Our results further demonstrated that primate *GBP1*, *GBP3* and *GBP7* are absent from the genome of muroids and showed that the *Gbp* gene annotations in muroids were incorrect. We propose a new classification based on the phylogenetic analyses and the divergence between the groups. Extrapolations to humans based on functional studies of muroid *Gbps* should be re-evaluated. The evolutionary analyses of muroid *Gbp* genes provided new insights about the evolution and function of these genes.

## Introduction

The innate ability of cells to resist against invading pathogens is due to the cell-autonomous immunity (1, 2). Upon the recognition of pathogens, production of type I interferon (IFN) and type II IFN increases, which results in the expression of numerous IFN-stimulated genes (3). Several of these genes enhance the efficacy of cell-autonomous immunity, including the guanylate binding proteins (GBPs), which are specialized in the host defense against intracellular pathogens ranging from bacteria to viruses (1, 4). The *Gbp* gene family belongs to the large dynamin GTPase superfamily that further includes myxoma resistance proteins, immunity-related GTPases proteins and the very large inducible GTPases (4). These proteins present structural and biochemical similarities (5, 6). The mammalian GBP vary from ~65 to 73 kDa in size and are mainly localized in the cytoplasm (4, 7).

Muridae and Cricetidae emerged 20 million years ago (Mya) from a single ancestor and are possibly the most successful mammals as they represent 27% of the total diversity (8). Muridae includes several subfamilies (Murinae, Lophiomyinae, Deomyinae and Gerbillinae) (9). Gerbillinae and Murinae appeared to have split ~17 Mya; however, the precise date still lack consensus (8). Within the Murinae group, rats and mice, diverged ~12.5 Mya (10). In Cricetidae, the first split occurred around 14.6 Mya, originating several subfamilies like Cricetinae, Arvicolinae and Neotominae (8). Muroids are scientifically important as they serve as a model for ecological and biomedical research (8, 11, 12). Further, they are hosts and vectors for many human diseases and evidence for co-speciation between rodents and viruses has been reported (13, 14). Despite its importance, there are some limitations in results’ extrapolation from the mouse model, considering inflammatory diseases, infection, sepsis and acute respiratory distress syndrome in humans (15, 16). Additionally, the phylogeny and the diversification patterns of such a relatively young group are not yet fully resolved, though several studies have been conducted (8, 12, 17, 18).

In mammals, *GBP* genes are usually organized in tandem on the same chromosome (19, 20). In primates, they are present on a single gene cluster (21), with humans presenting seven *GBPs* and one pseudogene located on chromosome 1 (1, 4). However, *Mus musculus Gbp* genes are found on two chromosomes, with *Gbp1*, *Gbp2*, *Gbp3 Gbp5* and *Gbp7* cluster together on chromosome 3, and chromosome 5 encodes *Gbp4*, *Gbp6*, *Gbp8*, *Gbp9*, *Gbp10* and *Gbp11* (4, 19).

In mice, GBPs can be induced by IFN, but it has been shown that interleukin (IL)-1α, IL-1β and tumor necrosis factor-α also induce the transcription of *Gbps* (22). Mouse GBP2, and possibly other GBPs from chromosome 3, has the ability to target vacuoles containing pathogens, like the *Salmonella typhimurium*, and promote the lysis of such vacuoles liberating the bacteria into the cytoplasm (4). Release of Gram-negative bacteria floods the cytoplasm with lipopolysaccharide (LPS), triggering the activation of NLRP3 inflammasome and leading to the production of pro-inflammatory cytokines, such as IL-1β and IL-18 (5, 7). As such, it was demonstrated that the knockout of *Gbp2* reduced IL-18 concentrations, which is crucial for IFN-γ-induced host defense against *Francisella novicida*, making mice highly susceptible to infections (5, 23, 24). In addition, mouse GBP2 has antiviral activity against vesicular stomatitis virus and encephalomyocarditis virus (25), the same as for human GBP1 (26, 27). Upon infection with pathogenic bacteria, mouse GBP5 regulates the activation of NLRP3 inflammasome (28). Both mouse GBP2 and GBP5 can independently control the pathways that promote AIM2 inflammasome activation during *F. novicida* infection (24). The deletion of *Gbp2*, *Gbp3*, *Gbp5* and *Gbp7* in mice leads to severe susceptibility for a broad range of pathogens and may also lead to different inflammatory phenotypes, in a similar manner to what occurs in human GBPs-defective cells (5). This highlights the importance of *Gbp* genes in the immune system of mammals.

Nei and Rooney (29) defined a multigene family as a group of genes that have originated from a common ancestral gene and present similar functions and DNA sequences. For several years, concerted evolution was invoked to explain the evolution of multigene families related to the immune system; however, this did not explain how some immune genes were more closely related between species than within the same species (30). Nei and colleagues proposed the birth-and-death model of evolution for genes of the immune system (30). Duplication of genes can be produced by tandem and gene-block duplication (30). Some duplicated genes may diverge, remaining functional, or even gain new functions, whereas others can suffer deleterious mutations, becoming pseudogenes, or can also be deleted from the genome (30). Considering that *Gbps* belong to a multigene family of the immune system and the existence of more than 200 orthologs, *Gbps* most likely follows the birth-and-death model of evolution (7, 21).

Over the past years, it has become clear that GBPs are major players of the host defense and are important against a broad array of pathogens (6, 31) making it relevant to study *Gbp* genes evolution and function. Despite this, only few studies have focused on the evolution and function of *Gbps* in muroids. As such, we investigated the *Gbp* multigene family in muroids to bring new insights about their evolution.

## Methods

### Phylogenetic Analysis

Complete coding sequences of *Gbps* were obtained from publicly available databases. We retrieved a total of 182 *Gbp* nucleotide sequences from 12 different species of Muridae and Cricetidae (124 sequences), *Homo sapiens* (7 sequences), *Tupaia glis* (5 sequences) and from 5 different species of primates (34 sequences) to increase the robustness of the analysis. *Loxodonta africana Gbps* were used as outgroups (12 sequences). Sequences were retrieved from species for which the genomes are available at GenBank and Ensembl (see **Supplementary Table 1** for accession numbers). Additionally, BLAST analyses were performed to confirm that all *Gbps* sequences were retrieved from the species used in this study. An alignment was performed in BioEdit (32) using Clustal W (33), followed by visual inspection. Before the phylogenetic analysis, the dataset was screened for gene conversion using GARD (Genetic Algorithm for Recombination Detection) (34).

Phylogenetic relationships among the *GBP* amino acid sequences were inferred in MEGA X (35) and RAxML (Randomized Axelerated Maximum Likelihood) v8.2.12 (35–37) using maximum-likelihood (ML) criteria, and with BEAST v1.10.4 for a Bayesian inference (38). The best-fit amino acid substitution model for *GBP* genes was determined in MEGA X and ProtTest v3.4.2 (39). In MEGA X, bootstrap (1000 replicates) was used to assess reliability and robustness of the phylogenetic tree branches. The best ML phylogeny was further determined using RAxML and branch supports were obtained using 1000 rapid bootstrap replicates as implemented in the method. In addition, two independent replicate runs of 10 million generations were performed in BEAST, using the Yule tree prior and an uncorrelated lognormal relaxed clock (40). The convergence of the BEAST runs was assessed using Tracer v1.7 (41); the resulting tree files were concatenated using LogCombiner v1.10.4, discarding the first 10% as burn-in, and posterior trees were summarized using TreeAnnotator v1.10.4, both included in the BEAST v1.10.4 package.

Sequence alignments can be found in **Supplementary Data**. Sequences that did not encode a putative functional protein, i.e. pseudogenes, were discarded from the analysis (see **Supplementary Table 1** for accession numbers).

### Genomic Synteny Analysis

NCBI (https://www.ncbi.nlm.nih.gov/genome/gdv/) and Ensembl (https://www.ensembl.org/index.html) were used to determine the relative syntenic positions and transcription orientation of *Gbps* across the genomes of Muridae and Cricetidae analyzed. BLAST analysis was performed to ensure that all *Gbp* genes of muroids were included in the study.

### Divergence Analyses

**Figure 1:**
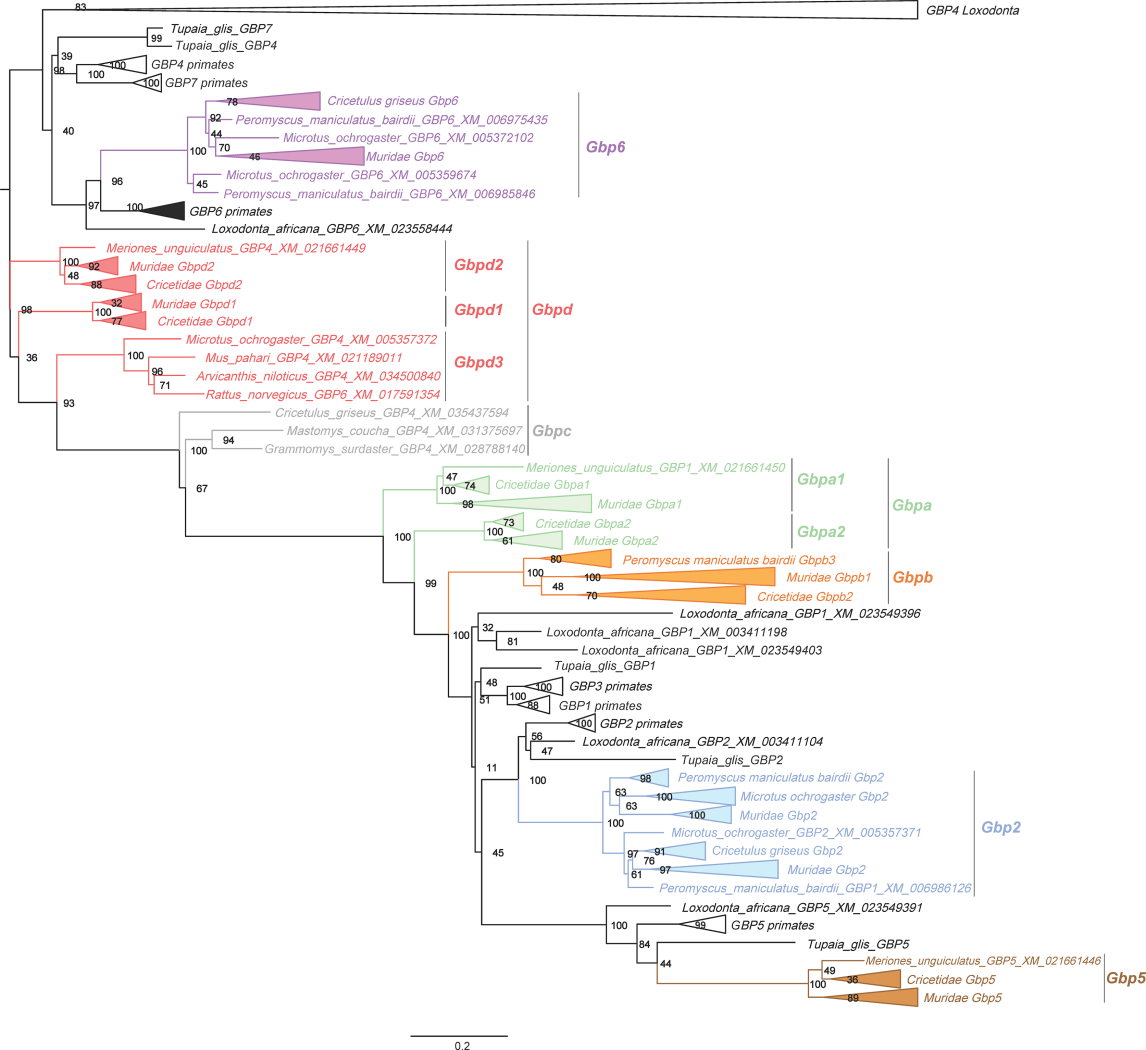
Randomized Axelerated Maximum Likelihood (RAxML) tree of the *Gbp* genes in Muridae and Cricetidae. The tree was obtained using the RAxML method using 1000 rapid bootstrap and is represented with midpoint root. Numbers on branches are the ML bootstrap values. All new *Gbpd* groups are also present. In **Supplementary Figure 1**, a full tree is depicted without collapsed nodes of Muridae and Cricetidae. Scale bar refers to the inferred amount of change per site along branches.

Genetic distances between the groups established based on the ML tree (see **Figure 1**) were calculated using MEGA X (35). Analyses were conducted using the JTT matrix-based model as determined by the same program (42). All ambiguous positions were removed for each sequence pair (pairwise deletion option). The analysis involved 165 amino acid sequences (*Tupaia glis* and *Loxodonta africana* sequences were not included) and there was a total of 676 amino acid positions in the final dataset.

## Results

### *Gbp* Phylogeny

A first screening of the dataset was performed using GARD to detect gene conversion/recombination. Overall, gene conversion was not detected (data not shown) and all sequences were included in the phylogenetic analyses. The obtained ML phylogenetic tree from RAxML (**Figure 1**, some Muridae and Cricetidae branches are collapsed, see **Supplementary Figure 1** for full tree) shows that primate and *Tupaia GBP1*, *GBP3* and *GBP7* are absent from the genomes of muroids as none of the retrieved Muridae and Cricetidae sequences clustered together with the corresponding *GBP*s from primates (**Figure 1**; **Supplementary Figures 1–3**). This suggests that the *Gbp* genes of muroids previously classified as *Gbp1*, *Gbp3* and *Gbp7* had been misclassified and that their reclassification is necessary. Furthermore, some *Gbps* within the major clusters also seem to have been misclassified. As such, we propose a new classification system for *Gbps* in Muridae and Cricetidae (see **Supplementary Table 2**). Following the obtained results, we propose a total of 87 changes; *Gbp5* classification remained unchanged.

From the analysis, muroids seem to encode a total of seven different *Gbps*. Muroid *Gbp2* (**Figure 1**, in blue) is present in all analyzed species. Duplication events seem to have occurred in *Gbp2* in most species with *Mus musculus*, *Mastomys coucha*, *Microtus ochrogaster* and *Cricetulus griseus* having two copies, *Mus caroli*, *Peromyscus maniculatus bairdii* and *Mus pahari* with three copies and *Arvicanthis niloticus* with four copies (**Figure 1** and **Supplementary Figure 1**). *Rattus norvegicus* and *Rattus rattus* have only one copy. *Gbp5* is present in all twelve species analyzed as a single copy gene. For *M. musculus*, two *Gbp5* sequences are available (*Gbp5* and *Gbp5a*; see **Supplementary Figure 1**). However, only one *Gbp*5 gene was detected in the genome. These two sequences are most likely allelic variations of *Gbp5* since the *M. musculus* genome has one of the highest genome sequencing coverages. Our ML tree results further indicate that rodent *Gbp2* (**Figure 1**, in blue) and *Gbp5* (**Figure 1**, in brown) are most likely orthologs of *GBP2* and *GBP5* from primates, with bootstrap values of 100 (**Figure 1**). In addition, some muroid *Gbps* within the *Gbp2* group seem to have also been misclassified (see **Supplementary Table 2**).

**Figure 2:**
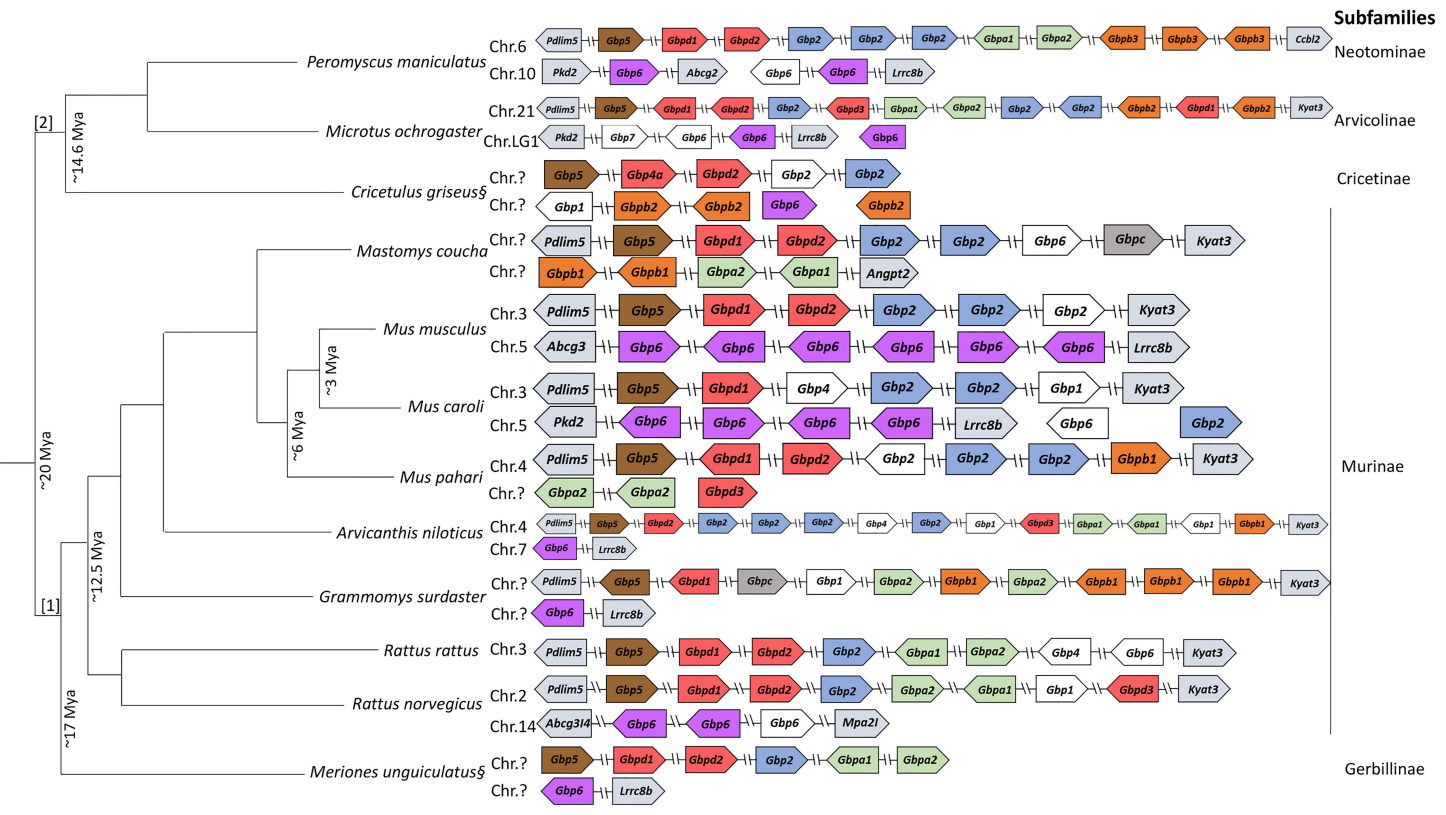
*Gbp* gene family synteny in Muridae and Cricetidae. Organization of the *Gbp* gene family in the species studied according to genomes available in NCBI (www.ncbi.nlm.nih.org) and Ensembl (wwww.ensembl.org). Arrows represent transcription orientation. White boxes indicate pseudogenes. The diagram is not to drawn to scale. Chromosomes are indicated when information is available. § Putative representation of the *Gbp* gene family in *Cricetulus griseus* and *Meriones unguiculatus*. [1]: Muridae; [2]: Cricetidae. Color scheme: 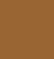 - *Gbp5*; 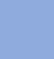 - *Gbp2*; 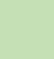 - *Gbpa*; 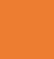 - *Gbpb*; 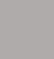
- *Gbpc*; 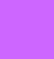
- *Gbp6*; 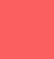 - *Gbpd*.

The *Gbp6* (**Figure 1**, in purple) is present in all families analyzed, suggesting it emerged in the common ancestor of Muridae and Cricetidae. It is only absent in *M. pahari* and *R. rattus*, while an expansion seems to have occurred in *M. caroli* and *M. musculus*, which have four and six copies, respectively (see **Supplementary Figure 1**). In the case of *M. musculus*, the *Gbp6* group includes the previously classified *Gbp4, 8, 9, 10* and *11* genes that are located on chromosome 5 (**Supplementary Table 2**). Indeed, all genes clustered in the *Gbp6* group are located on the second *Gbp* gene cluster (see **Figure 2**). Similarly, to *Gbp2* and *5*, *Gbp6* appears to be ortholog of primates *GBP6* clustering together with bootstrap value of 96 (**Figure 1** and **Supplementary Figures 1, 3**).

We identified four new groups of *Gbps* that appear to be only present in Muridae and Cricetidae and classified them as *Gbpa* (**Figure 1**, in green), *Gbpb* (**Figure 1**, in orange), *Gbpc* (**Figure 1**, in grey) and *Gbpd* (**Figure 1**, in red). These new groups are very-well supported with bootstrap values of ≥94 (**Figure 1**). *Gbpa* (**Figure 1**, in green) is divided into two highly supported subgroups, *Gbpa1* and *Gbpa2* (**Figure 1**; bootstrap value of 100). It seems that *Gbpa* duplicated in the common ancestor of Muridae and Cricetidae and evolved differently, originating the two *Gbpa* groups. The establishment of these two groups is also supported by the MEGAX ML tree and the BEAST analysis (see **Supplementary Figures 2.1, 3.2**, respectively). Indeed, several amino acid differences are found between them (see **Table 1**). For *M. pahari* and *Grammomys surdaster*, the two sequences are grouped in the *Gbpa2* group, which can be explained by an old duplication event followed by concerted evolution, however, we cannot exclude a recent duplication event in this species. Additionally, in three species (*M. musculus*, *M. caroli* and *C. griseus*), this gene was not detected in the genome, which suggests that it could have been independently deleted from the genome.

**Table 1:** Specific amino acids of the new *Gbps* of muroids.

Gene group	Characteristic amino acids	Position in alignment*	Domain
*Gbpa1*	QLQ	237 to 239	N-Terminal
*Gbpa1*	RDQALTV	580 to 586	C-Terminal
*Gbpa1*	HQERV	631 to 635	C-Terminal
*Gbpa2*	TLK	285 to 287	N-Terminal
*Gbpa2*	RIQLK	648 to 652	C-Terminal
*Gbpa2*	EGF	672 to 674	C-Terminal
*Gbpa2*	QEE	683 to 685	C-Terminal
*Gbpb1*	PCMES	412 to 416	C-Terminal
*Gbpb2*	SQTENA	422 to 427	C-Terminal
*Gbpb3*	WMWCVPHPQKSDHTLVLLDTEGLGDVEKG DNQNDCWIFALAVLLSSTFVYNSMGAINQQA	176 to 235	N-Terminal
*Gbpb3*	KFFPKKKCFVFERPAHGKKL	329 to 348	C-Terminal
*Gbpb3*	LVITYVNTIS	398 to 408	C-Terminal
*Gbpc*	DGQSLTADEYLENSLKLK	292 to 308	N-Terminal
*Gbpc*	LPGGIKVNGA	384 to 394	C-Terminal
*Gbpd1*	QKAME	588 to 592	C-Terminal
*Gbpd1*	KMETER	643 to 648	C-Terminal
*Gbpd2*	VTELTQLI	244 to 251	N-Terminal
*Gbpd2*	RYFFPVR	328 to 334	C-Terminal
*Gbpd2*	MEAQERSF	624 to 631	C-Terminal
*Gbpd3*	CITED	294 to 298	N-Terminal
*Gbpd3*	CIRQFFPRRKCFVF	326 to 339	C-Terminal
*Gbpd3*	WPVSDPQLL	341 to 349	C-Terminal

*The alignment is available in the **Supplementary Material**.

*Gbpb* is divided into three clusters, *Gbpb1*, *Gbpb2* and *Gbpb3* (bootstrap values of 100, 70 and 80 respectively, **Figure 1**, in orange). This gene seems to have been present before the split of Murinae and Cricetidae, but appears to be absent in *Meriones unguiculatus*, suggesting that it was deleted from the genome. *Gbpb1* includes only sequences from Murinae and is absent in the genus *Rattus*, in *M. musculus* and *M. caroli*. For *Grammomys surdaster*, four copies have been identified, which suggests that for this species several duplication events may have occurred. *Gbpb2* encompasses only sequences from Cricetidae (*Cricetulus griseus*, and *Microtus ochrogaster*; see **Supplementary Figure 1**, in orange). For *Cricetulus griseus*, five copies have been identified, also suggesting species-specific duplication events. Interestingly, *Gbpb3* is composed by three copies of *Peromyscus maniculatus bairdii*, this suggests that *Gbpb* has evolved independently in this species originating *Gbpb3* (**Supplementary Figure 1**).

The *Gbpc* group showed an unusual composition. In fact, by using RaxML analysis only two sequences appear clustered from Murinae species *Grammomys surdaster* and *Mastomys coucha*. However, using BEAST and ML all three sequences clustered together and are well supported (bootstrap 99; in grey; **Supplementary Figure 2.2** in grey) which indicates that *Gbpc* is composed of three sequences. The most likely explanation is the emergence of this gene in the ancestor of Muridae and Cricetidae family followed by a loss of this gene in most rodents species.

We consider that *Gbpd* from Muridae and Cricetidae is exclusive to these two families (**Figure 1**, in red). The *Gbpd* is divided into three groups that are well supported with bootstrap values of 100 (**Figure 1**, in red). It is composed of muroid sequences previously classified as *Gbp3, 4, 6* and *7* (see **Supplementary Figure 1**). These three groups include sequences from all muroids, suggesting that these three groups originated from an old duplication event that occurred before the radiation of Muridae and Cricetidae. The only exceptions are the absence of sequences from *Grammomys surdaster* and *M. caroli* in the *Gbpd2* group and the absence of *Arvicanthis niloticus* in *Gbpd1*. *Gbpd3* is composed of four sequences (*R. norvegicus*, *Microtus ochrogaster*, *Mus pahari* and *Arvicanthis niloticus*). We propose that this gene emerged in the ancestor of Muridae and Cricetidae family and was then lost in most rodents species.

### Synteny Analysis

Despite presenting similarities, muroids have a high plasticity regarding their *Gbps* synteny (**Figure 2**). Indeed, several rodent *Gbp* genes are clustered in more than one chromosome (e.g., *Mus musculus*, *Rattus norvegicus*, *Microtus ochrogaster* and *Mus caroli*). *Rattus rattus* seems to be an exception as it has, similar to primates, all *Gbp* copies in a single gene cluster on chromosome 3 (**Figure 2**).

Several duplication events are observed across all families, e.g., *Gbp6* in *M. musculus* and *M. caroli*, *Gbp2* in *M. musculus, M. caroli*, *M. pahari*, *Arvicanthis niloticus* and *Mastomys coucha*. Some genes seem to have lost their function and became pseudogenes, e.g., *Gbp2* in *Cricetulus griseus* and *M. pahari* and *M. musculus*. *Gbp5* and *Gbpd* appear to be the only genes from this multigene family that are present on the same location across each gene cluster from all species analyzed, adjacent to the *Pdlim5* gene (**Figure 2**). For the rest of this gene cluster, no specific patterns could be detected.

Considering the second cluster of *Gbp* genes, which is located on chromosome 5 in *M. musculus*, our results suggest that, at least for well-characterized species, the newly found genes belong to the *Gbp6* group. Since this occurred in all rodent species analyzed, this second gene cluster was most likely already existing in the common ancestor of these species. In *M. musculus* and *M. caroli*, a duplication event occurred in *Gbp6*. Surprisingly, this gene was not detected in several other species like *Mastomys coucha*, *M. pahari* and *R. rattus*. This might be explained by a genome deletion or a bad quality of the sequenced genomes (**Figure 2**).

### Divergence Analysis

Despite being phylogenetically close to their primate counterparts (**Figure 1**) and having a common ancestor, muroid and primates *GBP2*, *GBP5* and *GBP6* show high divergence values (~22%, ~30% and ~19%, respectively; **Table 2**). For instance, primates *GBP1* and *pGBP3* are considered as different groups with a divergence as low as 7% (**Table 2**). For muroids, a divergence of at least 21% exists between the different *Gbp* groups (**Table 2**). Considering that *Gbp* groups are well-supported in the phylogenetic trees (**Figure 1** and **Supplementary Figures 1–3**), several amino acid differences can be found between the muroid groups and several common amino acids can be identified within each established group in this work (see **Table 1**), our data support the newly proposed classification for muroid *Gbps*.

**Table 2:** Estimates of net evolutionary divergence between *Gbp* groups of sequences.

	Primates	Muridae and Cricetidae							Primates				
	*pGBP5*	*Gbp5*	*Gbp2*	*Gbpa*	*Gbpb*	*Gbpc*	*Gbp6*	*Gbpd*	*pGBP7*	*pGBP6*	*pGBP4*	*pGBP3*	*pGBP1*
***pGBP5***													
***Gbp5***	0,30												
***Gbp2***	0,42	0,48											
***Gbpa***	0,36	0,42	0,26										
***Gbpb***	0,36	0,43	0,27	0,21									
***Gbpc***	0,57	0,59	0,44	0,29	0,38								
***Gbp6***	0,72	0,75	0,60	0,59	0,58	0,39							
***Gbpd***	0,68	0,73	0,53	0,50	0,51	0,23	0,21						
***pGBP7***	0,70	0,76	0,59	0,55	0,59	0,36	0,31	0,20					
***pGBP6***	0,73	0,78	0,62	0,59	0,59	0,41	0,21	0,19	0,26				
***pGBP4***	0,67	0,73	0,56	0,52	0,54	0,32	0,23	0,13	0,14	0,19			
***pGBP3***	0,36	0,46	0,28	0,21	0,24	0,43	0,64	0,54	0,58	0,63	0,56		
***pGBP1***	0,36	0,46	0,26	0,19	0,24	0,43	0,64	0,54	0,59	0,63	0,55	0,07	
***pGBP2***	0,41	0,54	0,22	0,25	0,28	0,48	0,65	0,54	0,58	0,63	0,57	0,21	0,20

## Discussion

The *GBP* multigene family has an important role in the innate immune response against invading pathogens such as bacteria and viruses (1, 4). Rounds of duplication and the birth-and-death process shape the evolution of GBPs. Indeed, more than 200 GBPs orthologs have been described, but variable numbers of *GBP* genes exist in different species distributed in one or two chromosomal regions (7, 19). For example, in humans, seven *GBP* genes exist in a single cluster on chromosome 5, while in mice, 11 *Gbps* have been described scattered on two chromosomes, five on chromosome 3 and six on the second chromosome (19). Recently, we showed that *GBP7* genes are unique to primates and emerged following a duplication of *GBP4*, while *GBP3* is restricted to simians and originated from *GBP1* and *GBP6* duplicated in Tarsiiformes, with both copies remaining functional in Cebidae and Cercopithecidae (21).

The present study demonstrated that *Gbp3* and *Gbp7* are not present in rodents, consistent with our previous findings in primates (21). Additionally, *Gbp1* also appears to be absent from the muroid genomes, suggesting that other muroid *Gbps* might present similar biological activities. In contrast, *Gbp2*, *Gbp5* and *Gbp6* orthologs are present in Muridae and Cricetidae as confirmed by their clustering in the phylogenetic trees with primates *GBP2*, *GBP5* and *GBP6*, respectively (**Figure 1** and **Supplementary Figures 1–3**). This indicates that *Gbp2*, *Gbp5* and *Gbp6* were already present in the ancestor of rodents and primates at least ~96 Mya (43). Maintenance of these genes for such a long period of time might be explained by their importance in regulating the immune system against a broad range of pathogens. Indeed, GBP2 and GBP5 appear to have important roles against viral and bacterial infections and to induce immune responses in mouse and humans (24, 25, 28, 44). To our knowledge no studies have been performed about the function of mouse *Gbp6.*

Our ML phylogenetic tree strongly supports the existence of four new muroid *Gbp* groups and for which we suggest a new nomenclature as *Gbpa, Gbpb, Gbpc* and *Gbpd* (see **Supplementary Table 2**). All these groups are phylogenetically well-supported with high bootstrap values and show high levels of genetic divergence. Additionally, these groups present characteristic amino acids (**Table 1**). Interestingly, *Gbpa*, *Gbpb* and *Gbpc* are not present in *Mus musculus* and *M. caroli* genomes (**Figure 1**, highlighted in green, orange and grey, respectively). To our knowledge, these four new groups had not been described. This indicates that, within these groups, *Gbps* are poorly studied and incorrectly annotated. Indeed, several sequences previously classified as *Gbp1-7* were clustered in these new groups.

*Gbpa* appears to be present since the emergence of Muridae and Cricetidae (~20 Mya; 8); yet, *M. musculus*, *M. caroli* and *C. griseus* do not encode *Gbpa.* Thus, we hypothesize that evolutionary pressures led to its disappearance in these species. *Gbpb* emerged before the separation of Muridae with Cricetidae (~20 Mya) [**Figures 1** and **2**; (8)]. After their separation, *Gbpb* independently evolved in each family originating *Gbpb1* in Muridae and *Gbpb2* and *Gbpb3* in Cricetidae, the latest is only present in *Peromyscus maniculatus bairdii*. The *Gbpc* cluster is strongly supported (bootstrap value of 94) and has an amino acidic genetic distance of at least 23% from all the other groups (see **Table 2**). Interestingly, the *Gbpc* is present in only three species, two belonging to the Murinae (*Grammomys surdaster* and *Mastomys coucha*) and one to the Cricetinae (*Cricetulus griseus*). This evolutionary pattern is quite puzzling and two different hypotheses might explain it: i) an event of convergent evolution where the gene emerged independently in three different lineages, or, most likely, ii) it emerged in Muridae and Cricetidae at least ~20 Mya but was deleted from the genome in most of the rodent species. The *Gbp6* gene is widespread across all rodents in the second *Gbp* gene cluster (**Figure 2**), indicating that the second cluster emerged before the ancestor of Muridae and Cricetidae, as it clustered with primates *Gbp6*. Despite this, the gene is not present in the genome of *M. pahari*, *Mastomys coucha* and *R. rattus*. The second gene cluster could have been lost in these three species; however, further genome analyses are required since the genome might be poorly assembled. The *Gbp* evolution in the genus *Mus* shows some interesting features. Indeed, both *Gbpa* and *Gbpb* are not present in *M. musculus* and *M. caroli*, but exist in *M. pahari*, indicating that both genes were deleted from the genome after the divergence of *M. pahari* from *M. musculus* and *M. caroli* (~6 Mya) and the split between *M. musculus* and *M. caroli* around 3 Mya (10; **Figure 2**). The expansion of *Gbp6* in *M. musculus* (six copies) and *M. caroli* (four copies) might have been a strategy to compensate for the loss of *Gbpa* and *Gbpb*.

A fourth *Gbp* gene named in this work, *Gbpd*, it is divided into three well-supported groups designated as *Gbpd1*, *Gbpd2* and *Gbpd3*. All groups contain sequences from all species analyzed, which suggests that these three groups originated from an old duplication event that happened before the Muridae and Cricetidae radiation at least ~20 Mya. The only exceptions are the absence of *Gbpd2* in *Grammomys surdaster* and *M. caroli* and the absence of *Arvicanthis niloticus* in *Gbpd1*. *Gbpd3* sequences were only detected in *R. norvegicus*, *Arvicanthis niloticus* and *Mus pahari* (Muridae) and *Microtus ochrogaster* (Cricetidae), which suggests that this gene emerged in the ancestor of Muridae and Cricetidae family and was then lost in most muroid species.

The observed heterogeneity in the number of *Gbps* and gene copy numbers in Muridae and Cricetidae might be explained by a combination of: i) selective pressures in genes belonging to the immune system due to invading pathogens, that, as a consequence, drive host-specific adaptations and promote expansion and complexity of the immunological repertoire (5); ii) rodents have accelerated diversification rates which lead to the morphological, taxonomical, ecological and physiological diversity found within this group and allow them to explore and adapt to an array of different ecosystems being exposed to different environmental constraints (8, 11). In fact, the high divergence observed between the *Gbp* groups in Muridae and Cricetidae ranging from 21% to 75% and the emergence of five new *Gbp* unique to Muridae and Cricetidae support the high selective pressures imposed on this multigene family in muroids.

The number of genes in the major histocompatibility complex (MHC) and immunoglobulins (Ig) varies extensively across species, demonstrating that duplications and deletions are common in multigene families (29). This diversity is crucial in their function to defend the host against a broad range of invading pathogens that will act as the evolutionary pressure to promote the diversification of genes (29). Hence, gene duplication, mutation and diversifying selection are key mechanisms in the evolution of genes of the immune system (29). Our results suggest that the diversity found within *Gbps* is consistent with their role in triggering the host defense against various pathogens. Although some *Gbps* have become pseudogenes (**Figure 2**, in white) or have been lost (e.g. no *Gbp6* in *M. pahari* and *R. rattus* and no *Gbpa* and *Gbpb* in *M. caroli* and *M. musculus*), it is reasonable to consider that *Gbp* genes in muroids follow the birth-and-death model of evolution proposed by Nei and colleagues (30).

Finally, the presence of characteristic amino acids in each of the new proposed groups (**Table 2**) further supports their classification. Interestingly, most of these characteristic motifs were found downstream the amino acid position 307, which marks the beginning of the C-terminal domain or helical domain (45) where a post-translational modification (isoprenylation) can occur in human GBP 1, 2 and 5 and in mouse GBP2 and 5 (22, 46). This suggests that most GBPs are more conserved in their N-terminal, including their GTPase activity (45, 46). However, future studies should be conducted to assess the structure of these proteins and provide insights about their potential function.

In summary, and based on our results, the nomenclature of the *Gbp* multigene family in muroids requires an update. Indeed, and as noted by Vestal and Jeyaratnam, similar GBPs are not always the most closely related ones between species (22). The incorrect annotation of *Gbps* can be problematic, particularly since studies have evaluated the function of mouse *Gbps* by considering them as orthologs of the human genes. However, our phylogenetic results and estimated amino acid divergences suggest that many are not homologs to the human genes. As such, their biological functions might greatly differ from those of humans and translational studies using muroid *Gbps* might not have a correct biological meaning. Therefore, a new nomenclature, as the one proposed in this study, will lead to a proper *Gbp* gene annotation, specifically in muroids, and contribute to a better understanding of their evolution and function. Besides the different evolutionary patterns observed in this mammalian group, it is highly likely that most of them have an important function in the immune system. As such, new studies revealing the structural organization and new functional assays would bring new knowledge about the role of GBPs.

## Conclusion

Overall, rodents express seven different *Gbp* genes. *Gbp2*, *Gbp5* and *Gbp6* appear to be phylogenetically similar to their human counterparts. The primate *Gbp1*, *Gbp3* and *GBbp7* genes are not present in muroids. Four new *Gbp* genes exclusive to Muridae and Cricetidae were identified: *Gbpa*, *Gbpb, Gbpc* and *Gbpd*.

The distribution and number of *Gbp* genes across the different Muridae and Cricetidae genomes differs widely, with duplicated, deleted and pseudogenized genes. This indicates that the *Gbp* multigene family in muroids evolved under a very strong selective pressure with different evolutionary histories within and between the two muroid taxa.

Some muroid *Gbps* are phylogenetically different to those of humans and most likely have different functions. This means that translational studies from muroids to human should be re-evaluated. Additionally, this study provides new insight into the evolution of *Gbps* in muroids and demonstrates that *Gbp* genes in Muridae and Cricetidae have been poorly annotated. The new proposed classification better matches the evolution of *Gbps* in muroids and opens new research opportunities to study the evolution and function of the *Gbp* multigene family in rodents.

## Data Availability Statement

The original contributions presented in the study are included in the article/**Supplementary Material**. Further inquiries can be directed to the corresponding author.

## Author Contributions

JVC-R analyzed the data and wrote the manuscript. JM-F analyzed and discussed the data. H-MB and JA discussed the data. PE conceived the study, analyzed, and discussed the data. All authors edited the manuscript and approved the final draft.

## Funding

This work was funded by Fundação para a Ciência e Tecnologia (FCT) which supported JVC-R PhD fellowship (DFA/BD/4965/2020), the Assistant Researcher grant of JA (CEECIND/00078/2017), JM-F with the CEEC contract (CEECIND/00372/2018), the Principal Researcher grant of PE (CEECIND/01495/2020) and the project UIDB/50027/2020 (Base). H-MB acknowledges funding from the DFG (BA-6820/1-1).

## Conflict of Interest

The authors declare that the research was conducted in the absence of any commercial or financial relationships that could be construed as a potential conflict of interest.

## Publisher’s Note

All claims expressed in this article are solely those of the authors and do not necessarily represent those of their affiliated organizations, or those of the publisher, the editors and the reviewers. Any product that may be evaluated in this article, or claim that may be made by its manufacturer, is not guaranteed or endorsed by the publisher.
