# Supplementary material for: Evolution of Guanylate Binding Protein (GBP) Genes in Muroid Rodents (Muridae and Cricetidae) Reveals an Outstanding Pattern of Gain and Loss
Source: Front Immunol. 2022 Feb 9;13:752186. doi: 10.3389/fimmu.2022.752186 (PMC8863968; doi:10.3389/fimmu.2022.752186)
Supplement: Supplementary file 2 [file DataSheet_2.docx]

Supplementary Material

# Supplementary Table 1. Accession numbers of sequences included and excluded

|  | **Included sequences** |  |  |  |
| --- | --- | --- | --- | --- |
|  | Accession number | *Species* | *Gene name* |  |
| 1 | NM_002053 | *Homo sapiens* | *GBP1* |  |
| 2 | XM_012464810 | *Aotus nancymaae* | *GBP1* |  |
| 3 | XM_011956051 | *Colobus angolensis palliatus* | *GBP1* |  |
| 4 | XM_019026669 | *Gorilla gorilla gorilla* | *GBP1* |  |
| 5 | XM_001085311 | *Macaca mulatta* | *GBP1* |  |
| 6 | XM_001147994 | *Pan troglodytes* | *GBP1* |  |
| 7 | MK214685 | *Tupaia glis* | *GBP1* |  |
| 8 | XM_006233425 | *Rattus norvegicus* | *Gbp1* |  |
| 9 | XM_034500085 | *Arvicanthis niloticus* | *Gbp1* |  |
| 10 | XM_034500086 | *Arvicanthis niloticus* | *Gbp1* |  |
| 11 | XM_034500240 | *Arvicanthis niloticus* | *Gbp1* |  |
| 12 | XM_034500564 | *Arvicanthis niloticus* | *Gbp1* |  |
| 13 | XM_034500841 | *Arvicanthis niloticus* | *Gbp1* |  |
| 14 | XM_034500842 | *Arvicanthis niloticus* | *Gbp1* |  |
| 15 | XM_034500843 | *Arvicanthis niloticus* | *Gbp1* |  |
| 16 | XM_028788126 | *Grammomys surdaster* | *Gbp1* |  |
| 17 | XM_028788145 | *Grammomys surdaster* | *Gbp1* |  |
| 18 | XM_028788141 | *Grammomys surdaster* | *Gbp1* |  |
| 19 | XM_028788127 | *Grammomys surdaster* | *Gbp1* |  |
| 20 | XM_028788129 | *Grammomys surdaster* | *Gbp1* |  |
| 21 | XM_028788130 | *Grammomys surdaster* | *Gbp1* |  |
| 22 | XM_021196161 | *Mus pahari* | *Gbp1* |  |
| 23 | XM_021189400 | *Mus pahari* | *Gbp1* |  |
| 24 | XM_021189401 | *Mus pahari* | *Gbp1* |  |
| 25 | XM_032897242 | *Rattus rattus* | *Gbp1* |  |
| 26 | XM_032896554 | *Rattus rattus* | *Gbp1* |  |
| 27 | XM_032896553 | *Rattus rattus* | *Gbp1* |  |
| 28 | XM_021157847 | *Mus caroli* | *Gbp1* |  |
| 29 | XM_021154688 | *Mus caroli* | *Gbp1* |  |
| 30 | XM_031375692 | *Mastomys coucha* | *Gbp1* |  |
| 31 | XM_031342171 | *Mastomys coucha* | *Gbp1* |  |
| 32 | XM_031342170 | *Mastomys coucha* | *Gbp1* |  |
| 33 | XM_031342168 | *Mastomys coucha* | *Gbp1* |  |
| 34 | XM_031339220 | *Mastomys coucha* | *Gbp1* |  |
| 35 | XM_006986120 | *Peromyscus maniculatus bairdii* | *Gbp1* |  |
| 36 | XM_006986121 | *Peromyscus maniculatus bairdii* | *Gbp1* |  |
| 37 | XM_006986122 | *Peromyscus maniculatus bairdii* | *Gbp1* |  |
| 38 | XM_006986125 | *Peromyscus maniculatus bairdii* | *Gbp1* |  |
| 39 | XM_006986143 | *Peromyscus maniculatus bairdii* | *Gbp1* |  |
| 40 | XM_006986126 | *Peromyscus maniculatus bairdii* | *Gbp1* |  |
| 41 | XM_016004723 | *Peromyscus maniculatus bairdii* | *Gbp1* |  |
| 42 | XM_021661455 | *Meriones unguiculatus* | *Gbp1* |  |
| 43 | XM_021661450 | *Meriones unguiculatus* | *Gbp1* |  |
| 44 | XM_005357434 | *Microtus ochrogaster* | *Gbp1* |  |
| 45 | XM_005357374 | *Microtus ochrogaster* | *Gbp1* |  |
| 46 | XM_026784136 | *Microtus ochrogaster* | *Gbp1* |  |
| 47 | XM_026783891 | *Microtus ochrogaster* | *Gbp1* |  |
| 48 | XM_026784120 | *Microtus ochrogaster* | *Gbp1* |  |
| 49 | XM_005357436 | *Microtus ochrogaster* | *Gbp1* |  |
| 50 | XM_007653724 | *Cricetulus griseus* | *Gbp1* |  |
| 51 | XM_035438266 | *Cricetulus griseus* | *Gbp1* |  |
| 52 | XM_027385979 | *Cricetulus griseus* | *Gbp1* |  |
| 53 | XM_007607790 | *Cricetulus griseus* | *Gbp1* |  |
| 54 | XM_003513428 | *Cricetulus griseus* | *Gbp1* |  |
| 55 | NM_010259 | *Mus musculus* | *Gbp2b* |  |
| 56 | NM_010260 | *Mus musculus* | *Gbp2* |  |
| 57 | MK214686 | *Tupaia glis* | *GBP2* |  |
| 58 | XM_012464814 | *Aotus nancymaae* | *GBP2* |  |
| 59 | XM_011956052 | *Colobus angolensis palliatus* | *GBP2* |  |
| 60 | XM_019026761 | *Gorilla gorilla gorilla* | *GBP2* |  |
| 61 | XM_001085895 | *Macaca mulatta* | *GBP2* |  |
| 62 | XM_024346929 | *Pan troglodytes* | *GBP2* |  |
| 63 | NM_004120 | *Homo sapiens* | *GBP2* |  |
| 64 | NM_133624 | *Rattus norvegicus* | *Gbp2* |  |
| 65 | XM_021196037 | *Mus pahari* | *Gbp2* |  |
| 66 | XM_029538048 | *Mus pahari* | *Gbp2* |  |
| 67 | XM_029475563 | *Mus caroli* | *Gbp2* |  |
| 68 | XM_042280029 | *Peromyscus maniculatus bairdii* | *Gbp2* |  |
| 69 | XM_031375696 | *Mastomys coucha* | *Gbp2* |  |
| 70 | XM_021661452 | *Meriones unguiculatus* | *Gbp2* |  |
| 71 | XM_005357371 | *Microtus ochrogaster* | *Gbp2* |  |
| 72 | XM_027385428 | *Cricetulus griseus* | *Gbp2* |  |
| 73 | XM_003513953 | *Cricetulus griseus* | *Gbp2* |  |
| 74 | XM_007655145 | *Cricetulus griseus* | *Gbp2* |  |
| 75 | BC140837 | *Homo sapiens* | *GBP3* |  |
| 76 | XM_012464809 | *Aotus nancymaae* | *GBP3* |  |
| 77 | XM_011956056 | *Colobus angolensis palliatus* | *GBP3* |  |
| 78 | XM_004026097 | *Gorilla gorilla gorilla* | *GBP3* |  |
| 79 | XM_001083783 | *Macaca mulatta* | *GBP3* |  |
| 80 | XM_001146987 | *Pan troglodytes* | *GBP3* |  |
| 81 | NM_001289492 | *Mus musculus* | *Gbp3* |  |
| 82 | XM_006233426 | *Rattus norvegicus* | *Gbp3* |  |
| 83 | NM_052941 | *Homo sapiens* | *GBP4* |  |
| 84 | XM_012464811 | *Aotus nancymaae* | *GBP4* |  |
| 85 | XM_019027006 | *Gorilla gorilla gorilla* | *GBP4* |  |
| 86 | XM_009424854 | *Pan troglodytes* | *GBP4* |  |
| 87 | MK214687 | *Tupaia glis* | *GBP4* |  |
| 88 | XM_006233424 | *Rattus norvegicus* | *Gbp4* |  |
| 89 | XM_034500655 | *Arvicanthis niloticus* | *Gbp4* |  |
| 90 | XM_034500840 | *Arvicanthis niloticus* | *Gbp4* |  |
| 91 | XM_028760496 | *Grammomys surdaster* | *Gbp4* |  |
| 92 | XM_028788140 | *Grammomys surdaster* | *Gbp4* |  |
| 93 | XM_006534804 | *Mus musculus* | *Gbp4* |  |
| 94 | XM_021196297 | *Mus pahari* | *Gbp4* |  |
| 95 | XM_021189011 | *Mus pahari* | *Gbp4* |  |
| 96 | XM_021196276 | *Mus pahari* | *Gbp4* |  |
| 97 | XM_032897244 | *Rattus rattus* | *Gbp4* |  |
| 98 | XM_032897243 | *Rattus rattus* | *Gbp4* |  |
| 99 | XM_021158284 | *Mus caroli* | *Gbp4* |  |
| 100 | XM_031375697 | *Mastomys coucha* | *Gbp4* |  |
| 101 | XM_031375689 | *Mastomys coucha* | *Gbp4* |  |
| 102 | XM_031375686 | *Mastomys coucha* | *Gbp4* |  |
| 103 | XM_006986127 | *Peromyscus maniculatus bairdii* | *Gbp4* |  |
| 104 | XM_006986124 | *Peromyscus maniculatus bairdii* | *Gbp4* |  |
| 105 | XM_021661449 | *Meriones unguiculatus* | *Gbp4* |  |
| 106 | XM_021661447 | *Meriones unguiculatus* | *Gbp4* |  |
| 107 | XM_005357437 | *Microtus ochrogaster* | *Gbp4* |  |
| 108 | XM_005357372 | *Microtus ochrogaster* | *Gbp4* |  |
| 109 | XM_005357368 | *Microtus ochrogaster* | *Gbp4* |  |
| 110 | XM_026784137 | *Microtus ochrogaster* | *Gbp4* |  |
| 111 | XM_035437716 | *Cricetulus griseus* | *Gbp4* |  |
| 112 | XM_035437715 | *Cricetulus griseus* | *Gbp4* |  |
| 113 | XM_035437594 | *Cricetulus griseus* | *Gbp4* |  |
| 114 | NM_052942 | *Homo sapiens* | *GBP5* |  |
| 115 | XM_012464815 | *Aotus nancymaae* | *GBP5* |  |
| 116 | XM_004026101 | *Gorilla gorilla gorilla* | *GBP5* |  |
| 117 | XM_016922023 | *Pan troglodytes* | *GBP5* |  |
| 118 | MK214688 | *Tupaia glis* | *GBP5* |  |
| 119 | NM_153564 | *Mus musculus* | *Gbp5* |  |
| 120 | AF487898 | *Mus musculus* | *Gbp5a* |  |
| 121 | M81128 | *Mus musculus* | *Gbp5b* |  |
| 122 | NM_001108569 | *Rattus norvegicus* | *Gbp5* |  |
| 123 | XM_034500343 | *Arvicanthis niloticus* | *Gbp5* |  |
| 124 | XM_028760412 | *Grammomys surdaster* | *Gbp5* |  |
| 125 | XM_021196977 | *Mus pahari* | *Gbp5* |  |
| 126 | XM_032897245 | *Rattus rattus* | *Gbp5* |  |
| 127 | XM_021158401 | *Mus caroli* | *Gbp5* |  |
| 128 | XM_031375684 | *Mastomys coucha* | *Gbp5* |  |
| 129 | XM_016004731 | *Peromyscus maniculatus bairdii* | *Gbp5* |  |
| 130 | XM_021661446 | *Meriones unguiculatus* | *Gbp5* |  |
| 131 | XM_013350012 | *Microtus ochrogaster* | *Gbp5* |  |
| 132 | XM_007654340 | *Cricetulus griseus* | *Gbp5* |  |
| 133 | BC131713 | *Homo sapiens* | *GBP6* |  |
| 134 | XM_012464820 | *Aotus nancymaae* | *GBP6* |  |
| 135 | XM_012464776 | *Aotus nancymaae* | *GBP6* |  |
| 136 | XM_011956049 | *Colobus angolensis palliatus* | *GBP6* |  |
| 137 | XM_011956192 | *Colobus angolensis palliatus* | *GBP6* |  |
| 138 | XM_004026102 | *Gorilla gorilla gorilla* | *GBP6* |  |
| 139 | XM_015144533 | *Macaca mulatta* | *GBP6* |  |
| 140 | XM_015144556 | *Macaca mulatta* | *GBP6* |  |
| 141 | XM_016922096 | *Pan troglodytes* | *GBP6* |  |
| 142 | XM_011249385 | *Mus musculus* | *Gbp6* |  |
| 143 | XM_008770121 | *Rattus norvegicus* | *Gbp6* |  |
| 144 | XM_017591354 | *Rattus norvegicus* | *Gbp6* |  |
| 145 | XM_003749409 | *Rattus norvegicus* | *Gbp6* |  |
| 146 | XM_034508931 | *Arvicanthis niloticus* | *Gbp6* |  |
| 147 | XM_028782360 | *Grammomys surdaster* | *Gbp6* |  |
| 148 | XM_021162459 | *Mus caroli* | *Gbp6* |  |
| 149 | XM_029477690 | *Mus caroli* | *Gbp6* |  |
| 150 | XM_021162424 | *Mus caroli* | *Gbp6* |  |
| 151 | XM_021162399 | *Mus caroli* | *Gbp6* |  |
| 152 | XM_006985846 | *Peromyscus maniculatus bairdii* | *Gbp6* |  |
| 153 | XM_006975435 | *Peromyscus maniculatus bairdii* | *Gbp6* |  |
| 154 | XM_021647448 | *Meriones unguiculatus* | *Gbp6* |  |
| 155 | XM_005372102 | *Microtus ochrogaster* | *Gbp6* |  |
| 156 | XM_005359674 | *Microtus ochrogaster* | *Gbp6* |  |
| 157 | XM_035461023 | *Cricetulus griseus* | *Gbp6* |  |
| 158 | NM_207398 | *Homo sapiens* | *GBP7* |  |
| 159 | XM_012464812 | *Aotus nancymaae* | *GBP7* |  |
| 160 | XM_011956054 | *Colobus angolensis palliatus* | *GBP7* |  |
| 161 | XM_019027091 | *Gorilla gorilla gorilla* | *GBP7* |  |
| 162 | XM_015144520 | *Macaca mulatta* | *GBP7* |  |
| 163 | XM_009424758 | *Pan troglodytes* | *GBP7* |  |
| 164 | MK214689 | *Tupaia glis* | *GBP7* |  |
| 165 | NM_145545 | *Mus musculus* | *Gbp7* |  |
| 166 | XM_006535281 | *Mus musculus* | *Gbp8* |  |
| 167 | XM_006534924 | *Mus musculus* | *Gbp9* |  |
| 168 | NM_001039646 | *Mus musculus* | *Gbp10* |  |
| 169 | NM_001039647 | *Mus musculus* | *Gbp11* |  |
| 170 | XM_017599539 | *Rattus norvegicus* | *Gbp11* |  |
| 171 | XM_003411198 | *Loxodonta africana* | *GBP1* |  |
| 172 | XM_023549403 | *Loxodonta africana* | *GBP1* |  |
| 173 | XM_023549396 | *Loxodonta africana* | *GBP1* |  |
| 174 | XM_003411104 | *Loxodonta africana* | *GBP2* |  |
| 175 | XM_010591314 | *Loxodonta africana* | *GBP4* |  |
| 176 | XM_003411101 | *Loxodonta africana* | *GBP4* |  |
| 177 | XM_003411103 | *Loxodonta africana* | *GBP4* |  |
| 178 | XM_023549398 | *Loxodonta africana* | *GBP4* |  |
| 179 | XM_010591315 | *Loxodonta africana* | *GBP4* |  |
| 180 | XM_023549398 | *Loxodonta africana* | *GBP5* |  |
| 181 | XM_023448449 | *Loxodonta africana* | *GBP6* |  |
| 182 | XM_023558444 | *Loxodonta africana* | *GBP6* |  |
|  |  |  |  |  |
|  | **Excluded sequences** |  |  |  |
|  | Accession number | Species | Gene name | Reason excluded |
| 1 | XM_017591401 | *Rattus norvegicus* | *Gbp1* | No functional protein encoded |
| 2 | XM_035437310 | *Cricetulus griseus* | *Gbp1* | No functional protein encoded |
| 3 | XM_029475550 | *Mus caroli* | *Gbp1* | No functional protein encoded |
| 4 | XM_021154687 | *Mus caroli* | *Gbp1* | No functional protein encoded |
| 5 | XM_029534610 | *Mus pahari* | *Gbp1* | No functional protein encoded |
| 6 | XM_029537877 | *Mus pahari* | *Gbp1* | No functional protein encoded |
| 7 | XM_031342171 | *Mastomys coucha* | *Gbp1* | No functional protein encoded |
| 8 | XM_015991692 | *Peromyscus maniculatus bairdii* | *Gbp1* | No functional protein encoded |
| 9 | XM_006998702 | *Peromyscus maniculatus bairdii* | *Gbp1* | No functional protein encoded |
| 10 | XM_007655145 | *Cricetulus griseus* | *Gbp2* | No functional protein encoded |
| 11 | XM_007607910 | *Cricetulus griseus* | *Gbp2* | No functional protein encoded |
| 12 | XM_029534717 | *Mus pahari* | *Gbp2* | No functional protein encoded |
| 13 | XM_032896552 | *Rattus rattus* | *Gbp4* | No functional protein encoded |
| 14 | XM_021189316 | *Mus pahari* | *Gbp4* | No functional protein encoded |
| 15 | XM_029475483 | *Mus caroli* | *Gbp4* | No functional protein encoded |
| 16 | XM_015991265 | *Peromyscus maniculatus bairdii* | *Gbp4* | No functional protein encoded |
| 17 | XM_027441994 | *Cricetulus griseus* | *Gbp6* | No functional protein encoded |
| 18 | XM_016004403 | *Peromyscus maniculatus bairdii* | *Gbp6* | No functional protein encoded |
| 19 | XM_006250610 | *Rattus norvegicus* | *Gbp6* | No functional protein encoded |
| 20 | XM_021155127 | *Mus caroli* | *Gbp6* | No functional protein encoded |
| 21 | XM_031375698 | *Mastomys coucha* | *Gbp6* | No functional protein encoded |
| 22 | NC_045030 | *Mastomys coucha* | *Gbp6* | No functional protein encoded |
| 23 | XM_032896550 | *Rattus rattus* | *Gbp6* | Short sequence |
| 24 | XM_021154610 | *Mus caroli* | *Gbp6* | Short sequence |
| 25 | XM_026785037 | *Microtus ochrogaster* | *Gbp6* | No functional protein encoded |
| 26 | XM_029534655 | *Mus pahari* | *Gbp6* | No functional protein encoded |
| 27 | XM_029534654 | *Mus pahari* | *Gbp6* | No functional protein encoded |
| 28 | XM_026785036 | *Microtus ochrogaster* | *Gbp7* | No functional protein encoded |
