# Supplementary material for: Evolution of Guanylate Binding Protein (GBP) Genes in Muroid Rodents (Muridae and Cricetidae) Reveals an Outstanding Pattern of Gain and Loss
Source: Front Immunol. 2022 Feb 9;13:752186. doi: 10.3389/fimmu.2022.752186 (PMC8863968; doi:10.3389/fimmu.2022.752186)
Supplement: Supplementary file 3 [file DataSheet_3.docx]

# Supplementary Table 2. Proposed nomenclature for *Gbp* in Muridae and Cricetidae

|  | Accession number | Species | Gene name | New classification |
| --- | --- | --- | --- | --- |
| 1 | XM_034500085 | *Arvicanthis niloticus* | *Gbp1* | *Gbp2* |
| 2 | XM_034500086 | *Arvicanthis niloticus* | *Gbp1* | *Gbp2* |
| 3 | XM_034500240 | *Arvicanthis niloticus* | *Gbp1* | *Gbp2* |
| 4 | XM_034500564 | *Arvicanthis niloticus* | *Gbp1* | *Gbp2* |
| 5 | XM_006986126 | *Peromyscus maniculatus bairdii* | *Gbp1* | *Gbp2* |
| 6 | XM_026784136 | *Microtus ochrogaster* | *Gbp1* | *Gbp2* |
| 7 | XM_005357436 | *Microtus ochrogaster* | *Gbp1* | *Gbp2* |
| 8 | XM_006986125 | *Peromyscus maniculatus bairdii* | *Gbp1* | *Gbp2* |
| 9 | XM_031375692 | *Mastomys coucha* | *Gbp1* | *Gbp2* |
| 10 | XM_021157847 | *Mus caroli* | *Gbp1* | *Gbp2* |
| 11 | XM_021154688 | *Mus caroli* | *Gbp1* | *Gbp2* |
| 12 | XM_021196161 | *Mus pahari* | *Gbp1* | *Gbp2* |
| 13 | NM_010259 | *Mus musculus* | *Gbp2b* | *Gbp2* |
| 14 | XM_032897242 | *Rattus rattus* | *Gbp1* | *Gbp2* |
| 15 | XM_006233426 | *Rattus norvegicus* | *Gbp3* | *Gbpa1* |
| 16 | XM_032896554 | *Rattus rattus* | *Gbp1* | *Gbpa1* |
| 17 | XM_034500841 | *Arvicanthis niloticus* | *Gbp1* | *Gbpa1* |
| 18 | XM_031342171 | *Mastomys coucha* | *Gbp1* | *Gbpa1* |
| 19 | XM_021661450 | *Meriones unguiculatus* | *Gbp1* | *Gbpa1* |
| 20 | XM_005357434 | *Microtus ochrogaster* | *Gbp1* | *Gbpa1* |
| 21 | XM_016004723 | *Peromyscus maniculatus bairdii* | *Gbp1* | *Gbpa1* |
| 22 | XM_006986122 | *Peromyscus maniculatus bairdii* | *Gbp1* | *Gbpa2* |
| 23 | XM_005357374 | *Microtus ochrogaster* | *Gbp1* | *Gbpa2* |
| 24 | XM_021661455 | *Meriones unguiculatus* | *Gbp1* | *Gbpa2* |
| 25 | XM_021189400 | *Mus pahari* | *Gbp1* | *Gbpa2* |
| 26 | XM_021189401 | *Mus pahari* | *Gbp1* | *Gbpa2* |
| 27 | XM_032896553 | *Rattus rattus* | *Gbp1* | *Gbpa2* |
| 28 | XM_006233425 | *Rattus norvegicus* | *Gbp1* | *Gbpa2* |
| 29 | XM_031342170 | *Mastomys coucha* | *Gbp1* | *Gbpa2* |
| 30 | XM_034500842 | *Arvicanthis niloticus* | *Gbp1* | *Gbpa2* |
| 31 | XM_028788127 | *Grammomys surdaster* | *Gbp1* | *Gbpa2* |
| 32 | XM_028788130 | *Grammomys surdaster* | *Gbp1* | *Gbpa2* |
| 33 | XM_028788126 | *Grammomys surdaster* | *Gbp1* | *Gbpb1* |
| 34 | XM_028788129 | *Grammomys surdaster* | *Gbp1* | *Gbpb1* |
| 35 | XM_034500843 | *Arvicanthis niloticus* | *Gbp1* | *Gbpb1* |
| 36 | XM_028788145 | *Grammomys surdaster* | *Gbp1* | *Gbpb1* |
| 37 | XM_029538048 | *Mus pahari* | *Gbp2* | *Gbpb1* |
| 38 | XM_031339220 | *Mastomys coucha* | *Gbp1* | *Gbpb1* |
| 39 | XM_028788141 | *Grammomys surdaster* | *Gbp1* | *Gbpb1* |
| 40 | XM_031342168 | *Mastomys coucha* | *Gbp1* | *Gbpb1* |
| 41 | XM_006986120 | *Peromyscus maniculatus bairdii* | *Gbp1* | *Gbpb3* |
| 42 | XM_006986121 | *Peromyscus maniculatus bairdii* | *Gbp1* | *Gbpb3* |
| 43 | XM_006986143 | *Peromyscus maniculatus bairdii* | *Gbp1* | *Gbpb3* |
| 44 | XM_026783891 | *Microtus ochrogaster* | *Gbp1* | *Gbpb2* |
| 45 | XM_003513428 | *Cricetulus griseus* | *Gbp1* | *Gbpb2* |
| 46 | XM_026784120 | *Microtus ochrogaster* | *Gbp1* | *Gbpb2* |
| 47 | XM_007653724 | *Cricetulus griseus* | *Gbp1* | *Gbpb2* |
| 48 | XM_035438266 | *Cricetulus griseus* | *Gbp1* | *Gbpb2* |
| 49 | XM_027385979 | *Cricetulus griseus* | *Gbp1* | *Gbpb2* |
| 50 | XM_007607790 | *Cricetulus griseus* | *Gbp1* | *Gbpb2* |
| 51 | XM_028788140 | *Grammomys surdaster* | *Gbp4* | *Gbpc* |
| 52 | XM_031375697 | *Mastomys coucha* | *Gbp4* | *Gbpc* |
| 53 | XM_035437594 | *Cricetulus griseus* | *Gbp4* | *Gbpc* |
| 54 | XM_006535281 | *Mus musculus* | *Gbp8* | *Gbp6* |
| 55 | NM_001039647 | *Mus musculus* | *Gbp11* | *Gbp6* |
| 56 | M81128 | *Mus musculus* | *Gbp5b* | *Gbp6* |
| 57 | XM_006534804 | *Mus musculus* | *Gbp4* | *Gbp6* |
| 58 | XM_006534924 | *Mus musculus* | *Gbp9* | *Gbp6* |
| 59 | NM_001039646 | *Mus musculus* | *Gbp10* | *Gbp6* |
| 60 | XM_017599539 | *Rattus norvegicus* | *Gbp11* | *Gbp6* |
| 61 | XM_007655145 | *Cricetulus griseus* | *Gbp2* | *Gbp6* |
| 62 | NM_145545 | *Mus musculus* | *Gbp7* | *Gbpd1* |
| 63 | XM_021158284 | *Mus caroli* | *Gbp4* | *Gbpd1* |
| 64 | XM_021196297 | *Mus pahari* | *Gbp4* | *Gbpd1* |
| 65 | XM_028760496 | *Grammomys surdaster* | *Gbp4* | *Gbpd1* |
| 66 | XM_031375686 | *Mastomys coucha* | *Gbp4* | *Gbpd1* |
| 67 | XM_003749409 | *Rattus norvegicus* | *Gbp6* | *Gbpd1* |
| 68 | XM_032897244 | *Rattus rattus* | *Gbp4* | *Gbpd1* |
| 69 | XM_021661447 | *Meriones unguiculatus* | *Gbp4* | *Gbpd1* |
| 70 | XM_006986127 | *Peromyscus maniculatus bairdii* | *Gbp4* | *Gbpd1* |
| 71 | XM_035437716 | *Cricetulus griseus* | *Gbp4* | *Gbpd1* |
| 72 | XM_005357368 | *Microtus ochrogaster* | *Gbp4* | *Gbpd1* |
| 73 | XM_005357437 | *Microtus ochrogaster* | *Gbp4* | *Gbpd1* |
| 74 | XM_017591354 | *Rattus norvegicus* | *Gbp6* | *Gbpd3* |
| 75 | XM_034500840 | *Arvicanthis niloticus* | *Gbp4* | *Gbpd3* |
| 76 | XM_021189011 | *Mus pahari* | *Gbp4* | *Gbpd3* |
| 77 | XM_005357372 | *Microtus ochrogaster* | *Gbp4* | *Gbpd3* |
| 78 | XM_006986124 | *Peromyscus maniculatus bairdii* | *Gbp4* | *Gbpd2* |
| 79 | XM_035437715 | *Cricetulus griseus* | *Gbp4* | *Gbpd2* |
| 80 | XM_026784137 | *Microtus ochrogaster* | *Gbp4* | *Gbpd2* |
| 81 | XM_021661449 | *Meriones unguiculatus* | *Gbp4* | *Gbpd2* |
| 82 | XM_034500655 | *Arvicanthis niloticus* | *Gbp4* | *Gbpd2* |
| 83 | NM_001289492 | *Mus musculus* | *Gbp3* | *Gbpd2* |
| 84 | XM_021196276 | *Mus pahari* | *Gbp4* | *Gbpd2* |
| 85 | XM_031375689 | *Mastomys coucha* | *Gbp4* | *Gbpd2* |
| 86 | XM_006233424 | *Rattus norvegicus* | *Gbp4* | *Gbpd2* |
| 87 | XM_032897243 | *Rattus rattus* | *Gbp4* | *Gbpd2* |
