# Supplementary material for: Evolution of Guanylate Binding Protein (GBP) Genes in Muroid Rodents (Muridae and Cricetidae) Reveals an Outstanding Pattern of Gain and Loss
Source: Front Immunol. 2022 Feb 9;13:752186. doi: 10.3389/fimmu.2022.752186 (PMC8863968; doi:10.3389/fimmu.2022.752186)
Supplement: Supplementary file 4 [file DataSheet_4.docx]

Supplementary Material


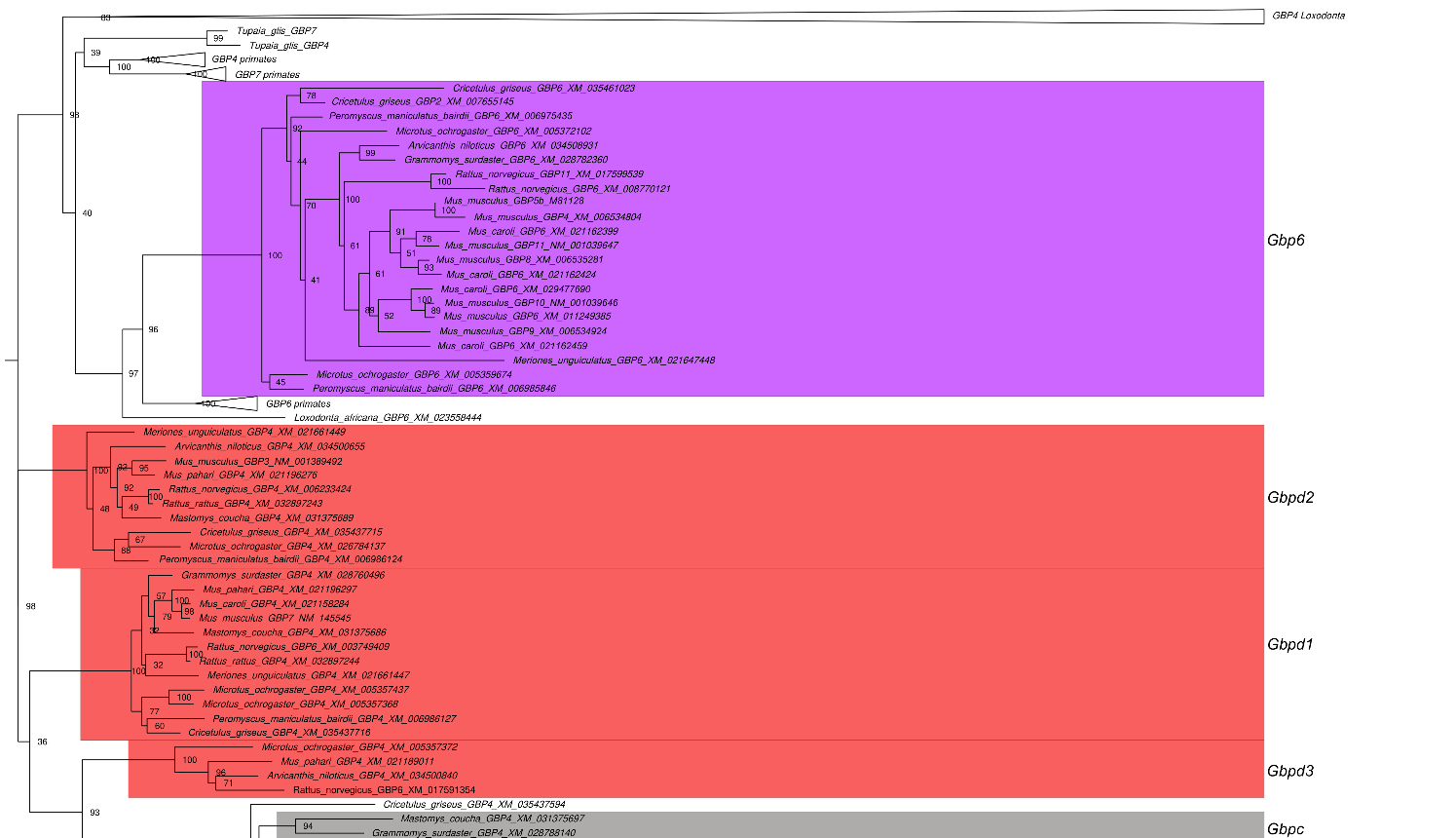


**Supplementary Figure 1.1** **Randomized Axelerated Maximum Likelihood (RAxML) tree of the *Gbp* genes in rodents Muridae and Cricetidae.** The tree was obtained using the RAxML method using 1000 rapid bootstrap and is represented with midpoint root. Numbers on branches are the ML bootstrap values. This part contains primate *GBP4* and *7*, *Gbp6* clusters with primates *GBP6*. All new *Gbpd* groups are also present**.**


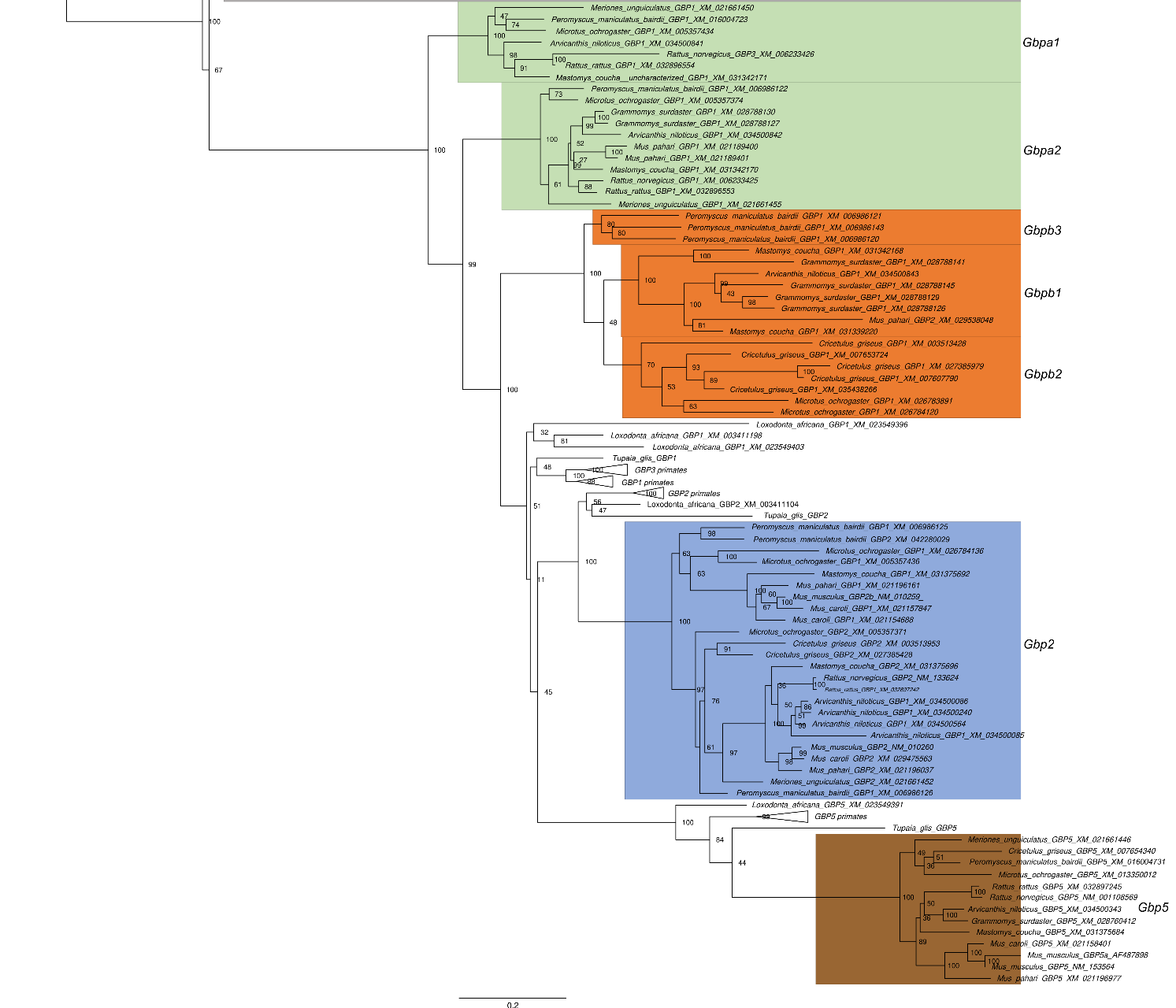


**Supplementary Figure 1.2** **Randomized Axelerated Maximum Likelihood (RAxML) tree of the *Gbp* genes in Muridae and Cricetidae.** The tree was obtained using the RAxML method using 1000 rapid bootstrap and is represented with midpoint root. Numbers on branches are the ML bootstrap values. This part contains *Gbpa*, *Gbpb* and *Gbpc***.** Scale bar refers to the inferred amount of change per site along
