# Supplementary material for: Evolution of Guanylate Binding Protein (GBP) Genes in Muroid Rodents (Muridae and Cricetidae) Reveals an Outstanding Pattern of Gain and Loss
Source: Front Immunol. 2022 Feb 9;13:752186. doi: 10.3389/fimmu.2022.752186 (PMC8863968; doi:10.3389/fimmu.2022.752186)
Supplement: Supplementary file 5 [file DataSheet_5.docx]

Supplementary Material


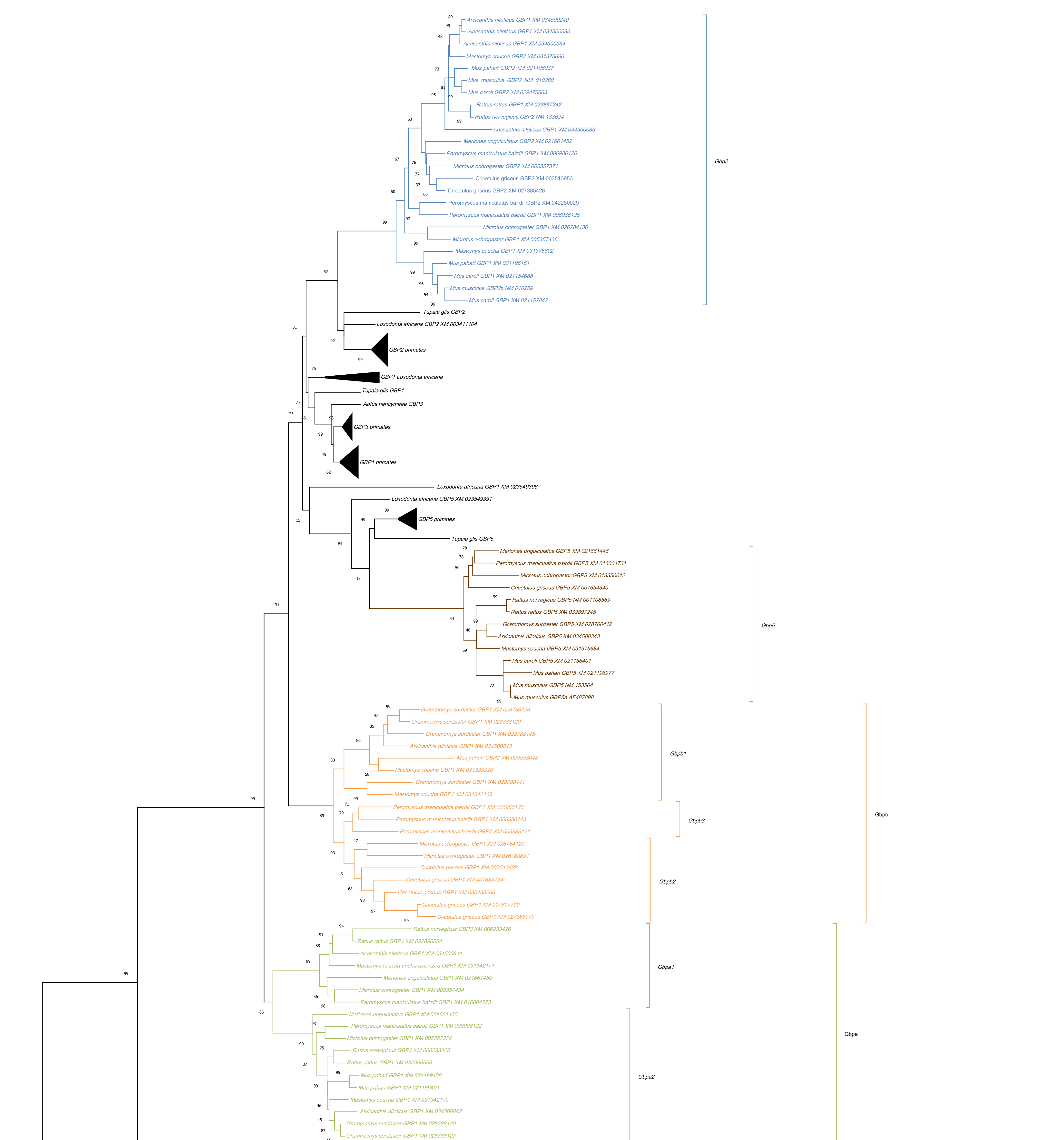


# Supplementary Figure 2.1 Maximum Likelihood (ML) tree of the *Gbp* genes in Muridae and Cricetidae. The tree was obtained with MEGA X (Kumar et al., 2018). The tree is drawn to scale, with branch lengths measured in the number of amino acid substitutions per site. The Jones-Taylor-Thornton model was used as determined by MEGA X (Kumar et al., 2018) and all positions with less than 95% site coverage were eliminated. The percentage of trees in which the associated taxa clustered together was determined from 1000 bootstrap replicates and is shown next to the branches. Contains muroid sequences from *Gbp 2*, *5*, *a* and *b*. Primates *GBP1, 2, 3* and *5* are also depicted.

#
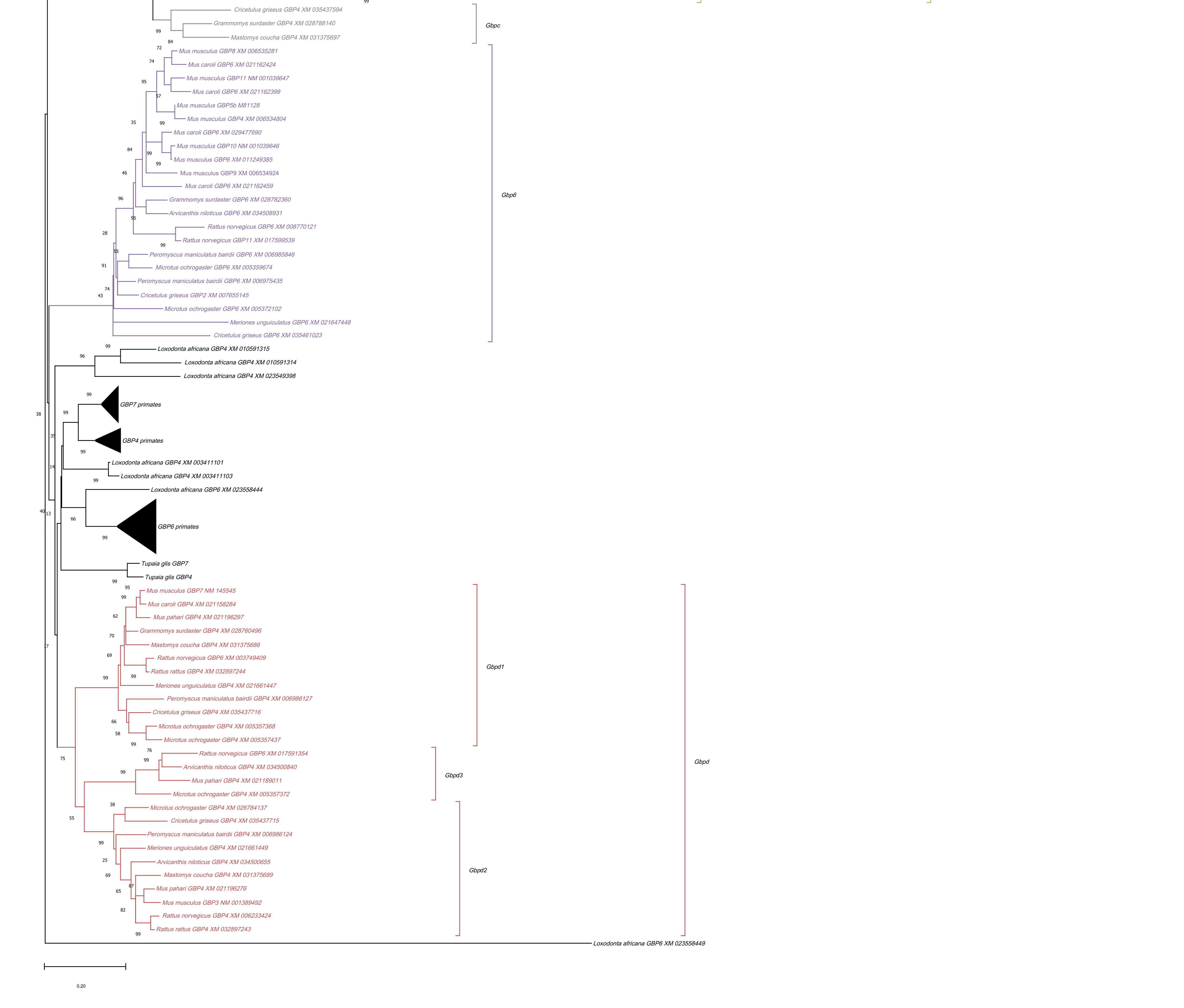


# Supplementary Figure 2.2 Maximum Likelihood (ML) tree of the *Gbp* genes in Muridae and Cricetidae. The tree is drawn to scale, with branch lengths measured in the number of amino acid substitutions per site. The percentage of trees in which the associated taxa clustered together was determined from 1000 bootstrap replicates and is shown next to the branches. Muroid *Gbpc, 6* and *d* are present.
