# Supplementary material for: Evolution of Guanylate Binding Protein (GBP) Genes in Muroid Rodents (Muridae and Cricetidae) Reveals an Outstanding Pattern of Gain and Loss
Source: Front Immunol. 2022 Feb 9;13:752186. doi: 10.3389/fimmu.2022.752186 (PMC8863968; doi:10.3389/fimmu.2022.752186)
Supplement: Supplementary file 6 [file DataSheet_6.docx]

Supplementary Material


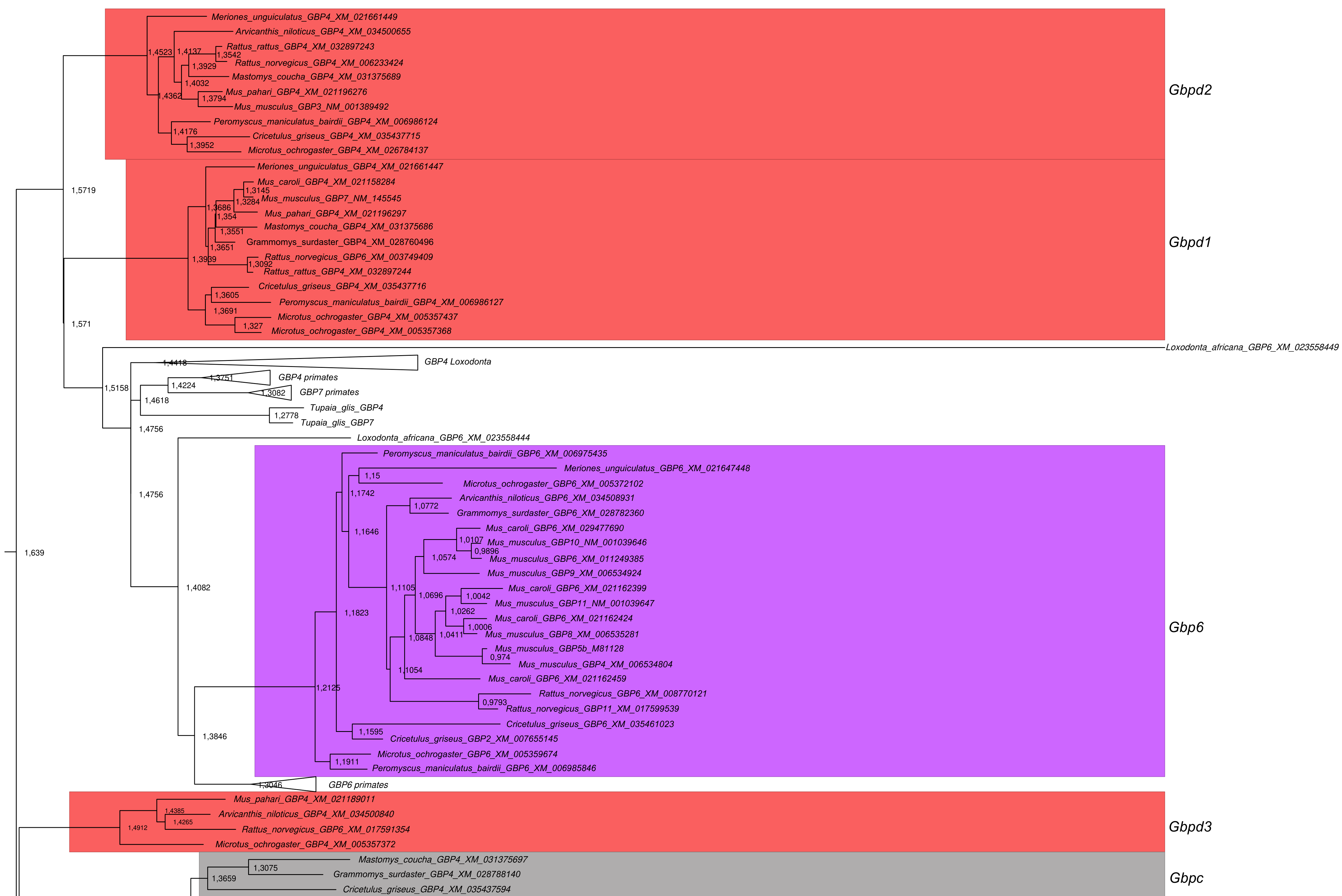


# Supplementary Figure 3.1. Bayesian inference of *Gbp* phylogeny in Muridae and Cricetidae. The tree is drawn to scale with branch length measuring the number of amino acid substitutions per site and using the midpoint root criterion. The model used for BEAST analysis was JTT+G+I+F (gamma model + proportion of invariant sites + empirical frequencies). This part contains *Gbpd* (red)*, c* (grey) and *Gbp6* (purple), additionally sequences of *GBP4*, *6* and *7* of primates are also present.


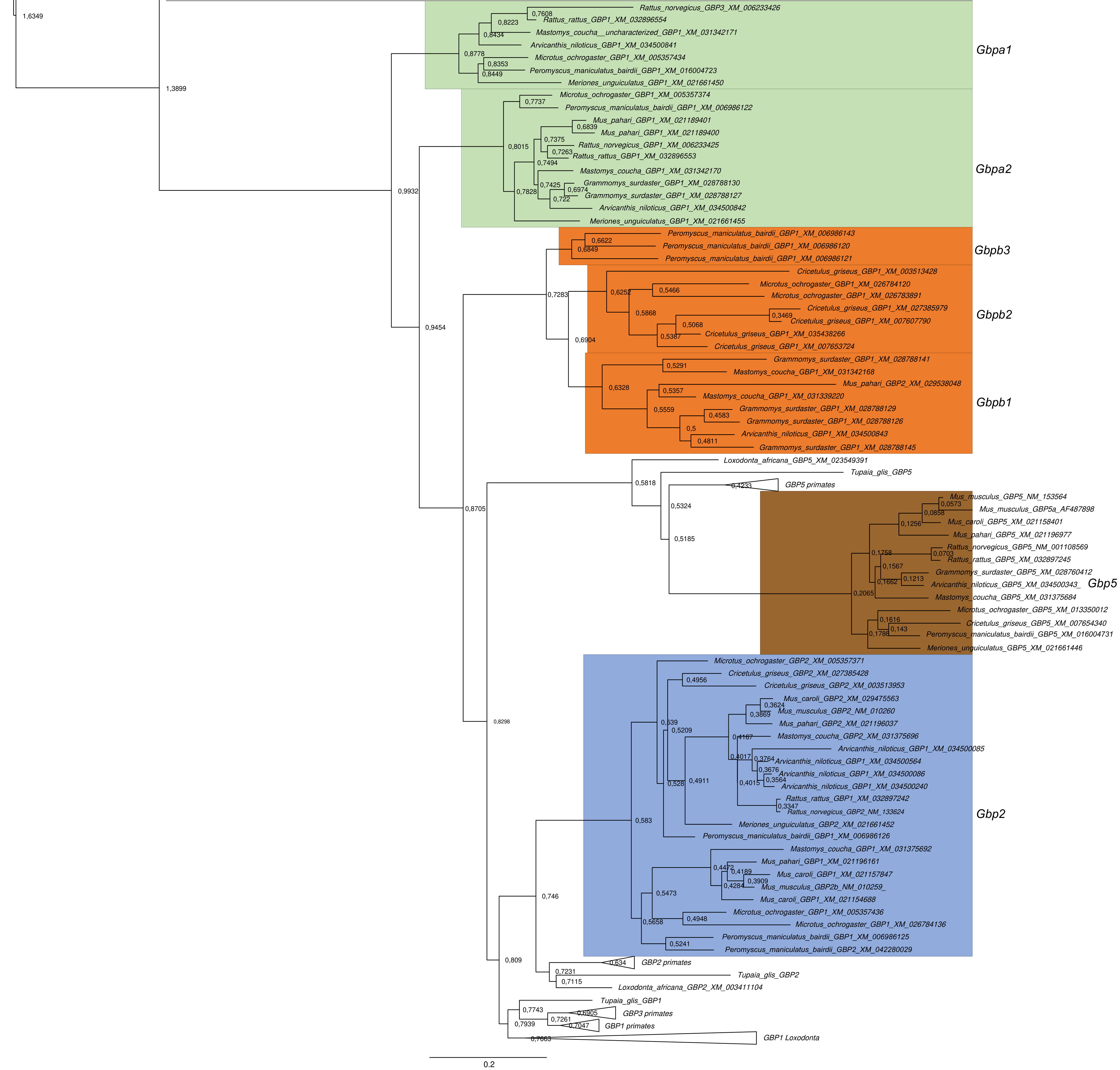


**Supplementary Figure 3.2. Bayesian inference of *Gbp* phylogeny in Muridae and Cricetidae.** The tree is drawn to scale with branch length measuring the number of amino acid substitutions per site and using the midpoint root criterion. This part contains *Gbpa* (green), *Gbpb* (orange), *Gbp2* (blue) and *Gbp5* (brown). Primates *GBP2* and *5* are also depicted.
